# Iron deficiency and changes in sleep: two conditions that compromise child growth and development

**DOI:** 10.1590/1984-0462/2025/43/2024017

**Published:** 2024-06-14

**Authors:** Luiz Antonio Del Ciampo, Ieda Regina Lopes Del Ciampo

**Affiliations:** a Universidade de São Paulo – Ribeirão Preto, SP, Brazil.; b Universidade Federal de São Carlos, São Carlos, SP, Brazil.

The article by Rodrigues Junior et al. addresses two frequent and important aspects that are often neglected in infant health care: iron deficiency anemia and sleep hygiene.^1^ The study revealed that children with anemia were more likely to have short sleep duration, demonstrating that the concomitance of these factors can compromise the development of the central nervous system.

Iron is involved in many fundamental processes in the brain such as oxygen transport, DNA synthesis, mitochondrial respiration, myelin synthesis and neurotransmitter metabolism.^2^ Iron deficiency causes changes in the hippocampus, corpus striatum, and neurotransmitters such as serotonin, dopamine, and noradrenaline. The hippocampus is essential for memory and learning, being very sensitive to a lack of iron. The corpus striatum controls activities such as attention, planning, emotion regulation, memory storage and retrieval, motivation, and reward.^3^ Therefore, iron deficiency during childhood can cause damage to the nervous tissue and the function of neurotransmitters and has been related to impairments in neurocognitive development (memory, attention, learning) and behavioral disorders.^4^

Sleep is an essential physiological function of the human body and presents a circadian sleep-wake rhythm that oscillates every 24 hours, serving to ensure brain health, including memory consolidation, retention of learning, vocabulary, emotional processing, maintenance of neural networks and synaptic plasticity, in addition to the regulation of several hormones.^5^

Not getting enough sleep can cause irritability, lack of interest and reduced skills in daily activities, as well as difficulty concentrating. Reducing sleep time can also lead to a decrease in GH levels, changes in the production of leptin and TSH, as well as problems with the immune system.^6^

Given this evidence, it is necessary to encourage puericulture and childcare health services as fundamental actions to improve children’s care and health conditions. In each routine consultation, possible factors that can lead to low iron concentration (prematurity, lack of breastfeeding, inadequate introduction of food, spoliation by parasites or infections) must be rigorously checked, also observing the guidelines for supplementation of the mineral in dosage and determined times. Furthermore, sleep hygiene must be properly guided considering the child’s needs, environmental conditions with adequate temperature and ventilation, reduction of sound and light stimuli, use of appropriate clothing and correct positioning of the child in bed.
